# Reconsideration of the transoral odontoidectomy in complex craniovertebral junction patients with irreducible anterior compression

**DOI:** 10.1186/s41016-020-00210-4

**Published:** 2020-09-15

**Authors:** Xingwen Wang, Longbing Ma, Zhenlei Liu, Zan Chen, Hao Wu, Fengzeng Jian

**Affiliations:** Department of Neurological Surgery, Xuanwu Hospital, Capital Medical University, Beijing, 100053 China

**Keywords:** Transoral approach, Odontoidectomy, Basilar invagination, Atlantoaxial dislocation, Image-guided navigation

## Abstract

**Background:**

Although the single-stage posterior realignment craniovertebral junction (CVJ) surgery could treat most of the basilar invagination (BI) and atlantoaxial dislocation (AAD), there are still some cases with incomplete decompression of the spinal cord, which remains a technique challenging situation.

**Methods:**

Eleven patients were included with remained myelopathic symptoms after posterior correction due to incomplete decompression of the spinal cord. Transoral odontoidectomy assisted by image-guided navigation and intraoperative CT was performed. Clinical assessment and image measurements were performed preoperatively and at the most recent follow-up.

**Results:**

Eleven patients were followed up for an average of 47 months. Symptoms were alleviated in 10 of 11 patients (90.9%). One patient died of an unknown reason 1 week after the transoral approach. The clinical and radiological parameters pre- and postoperatively were reported.

**Conclusion:**

Transoral odontoidectomy as a salvage surgery is safe and effective for properly selected BI and AAD patients after inadequate indirect decompression from posterior distraction and fixation. Image-guided navigation and intraoperative CT can provide precise information and accurate localization during operation, thus enabling complete resection of the odontoid process and decompression of the spinal cord.

## Background

Basilar invagination (BI) is the most common congenital malformation of the craniovertebral junction (CVJ), and it is characterized by protrusion of the odontoid process into the foramen magnum leading to ventral compression of the spinal cord [1–5]. BI is often associated with atlantoaxial dislocation (AAD), atlas assimilation, Chiari malformation, and other congenital anomalies [6, 7]. For the treatment of BI, direct transoral decompression followed by posterior instrumentation had traditionally been a standard treatment for decades [3, 4, 8–11]. Over the past decade, however, single-stage posterior realignment CVJ surgery to treat BI with AAD has been widely accepted as first-line treatment for this pathological entity, usually obviating the need for a transoral odontoidectomy. Good clinical and radiological outcomes have been reported in most patients with posterior-only treatment [4, 12–14]. However, a small proportion of patients continue to have persistent myelopathy because of incomplete decompression of the spinal cord after the index surgery. A second-stage transoral odontoidectomy to completely decompress the spinal cord is a salvage procedure [13]. Considering the complex bony abnormal configuration and the low overall incidence of this entity, transoral odontoidectomy is still technically demanding [8, 11, 15, 16].

In this article, we describe our experience in the treatment of 11 patients with BI and AAD who had undergone incomplete decompression of spinal cord or continued neurological deterioration following posterior distraction and fixation.

## Methods

This retrospective study was conducted under the approval of the ethics committee board of our hospital. Informed consent was obtained from each patient.

### Patient population and exclusion criteria

This retrospective analysis included BI and AAD patients who were managed at the Department of Neurological Surgery, Xuanwu Hospital, between December 2011 and March 2018. Out of the 135 treated patients, there were 11 patients with AAD and BI who had continued to have myelopathic symptoms after posterior fixation and distraction. Patients were excluded if they had any of the following: (1) rheumatoid arthritis, (2) OS odontoideum, (3) trauma at the CVJ, (4) infection, (5) osteoporosis, or (6) tumor.

### Clinical and radiographic assessment

There were 3 males and 8 females with a mean age of 42.7 years (range from 27 to 66). The demographic and clinical information is shown in Table 1. All 11 patients were treated by transoral odontoidectomy with image-guided navigation (Brainlab AG, Munich, Germany) and intraoperative CT (O-arm, Medtronic Memphis, TN, USA). Residual myelopathy due to incomplete spinal cord decompression or progressive neurological deterioration following the primary procedure was the main reason for secondary surgery. After the index surgery, radiographic imaging with CT and MRI confirmed incomplete or no reduction of the dontoid process, with persistent odontoid impingement on the spinal cord, and thus, a salvage anterior transoral odontoidectomy was performed in these patients. Pre- and postoperative Japanese Orthopedic Association (JOA) scores were collected to evaluate the clinical status before and after the surgery. Pre- and postoperative cervicomedullary angles (CMA) were measured to evaluate spinal cord decompression [17]. CMA was measured as the angle between the ventral part of the upper cervical spinal cord and the ventral part of the medulla oblongata on the sagittal MRI.

**Table 1:** Clinical features, demographic data and the pre- and postoperative JOA scale, CMA, operation time and blood loss

Pt	Sex	Age (y/o)	Diagnosis	Duration from first operation (y)	Follow-up (m)	JOA (pre)	JOA (post)	CMA (pre)	CMA (post)	Op time (min)	Blood loss (ml)
1	F	62	BI/AAD	5	76	7	11	120	127	170	50
2	F	47	BI/AAD, Chiari malformation, syringomyelia	2.5	47	8	13	109	125	200	50
3	F	66	BI/AAD, syringomyelia	14	26	6	11	114	126	198	100
4	M	31	BI/AAD, Chiari malformation	1	20	8	10	131	135	160	100
5	F	38	BI/AAD	0.25	81	6	10	103	117	80	200
6	F	27	BI/AAD, Klippel-Feil syndrome	5	47	6	11	95	109	209	20
7	M	31	BI/AAD, syringomyelia	3	18	8	14	127	132	200	50
8	F	39	BI/AAD, syringomyelia	8	76	7	10	130	142	165	50
9	F	45	BI/AAD, os odontoidem	0.3	59	7	13	145	149	185	150
10	M	39	BI/AAD	1.5	4	6	10	114	125	160	65
11	F	45	BI/AAD	2	8	8	12	124	133	188	106

### Statistical analysis

SPSS 19.0 statistic software (SPSS Inc., Chicago, Illinois, USA) was used for all statistical analyses in this study. Descriptive data are represented as means ± standard deviation. Clinical and radiological data pre-, postoperative were compared using the paired *t* test. A *p* value of less than .05 was considered to be statistically significant.

#### Surgical procedure

Before the transoral approach, preoperative CT images were obtained to register the navigation system.

**Fig. 1:**
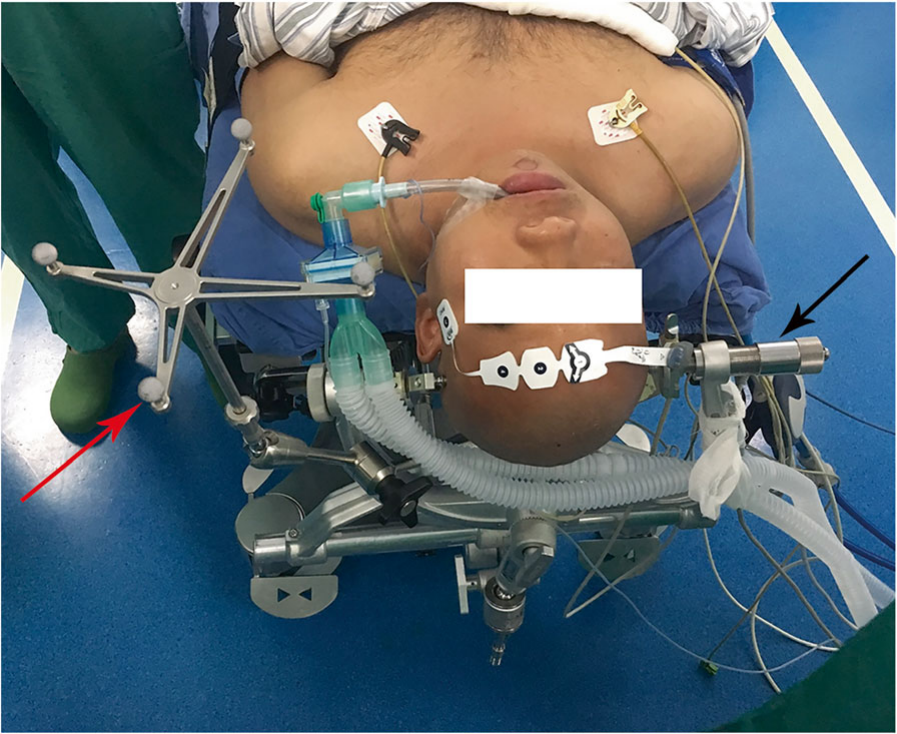
Patient position and navigation assembly. The red arrow indicates reference frame

**Fig. 2:**
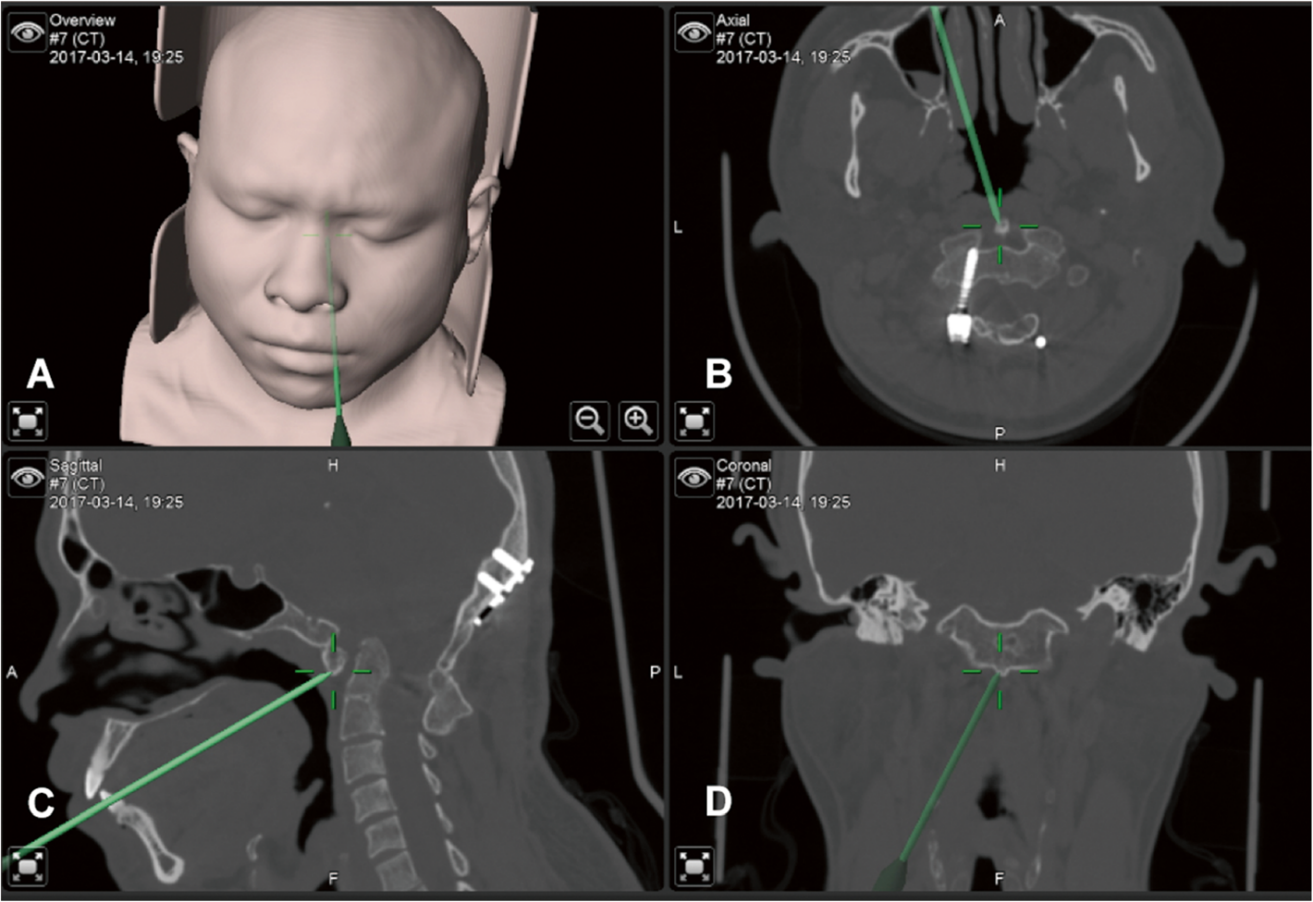
The incision on the posterior pharyngeal wall, anatomical landmarks, and the resection range of the odontoid process were determined according to the navigation. **a** Facial surface registration. **b**, **c**, **d** Axial, sagittal, coronal CT navigation image indicating the midpoint of anterior C1 arch lower margin

**Fig. 3:**
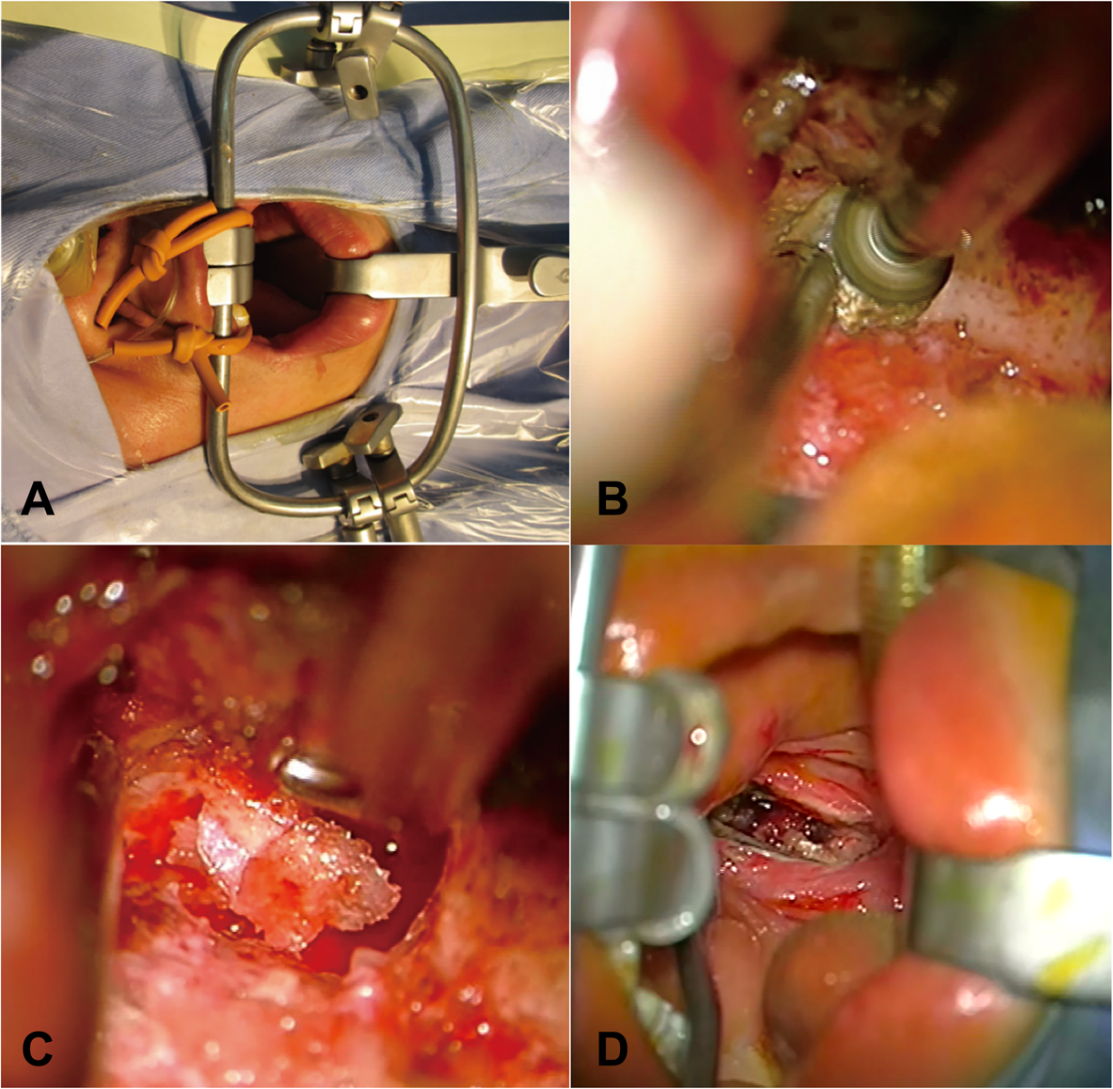
**a** The Davis mouth gag was placed and bilateral catheters were ligated to expose the posterior pharyngeal wall. **b** Using a diamond microdrill to remove the C2 vertebral body. **c** Gently removing the residual bony strip with a Kerrison to expose the dura mater. **d** The dura mater was clearly exposed and the odontoid process was completely resected

After general anesthesia via endotracheal intubation, the patient was placed in a supine position with the neck slightly extended to gain a better exposure of the odontoid process. The head was fixed with the Mayfield fixation pins, and the table was positioned to maintain the face in a horizontal position (Fig. 1). The reference frame for the navigation system was firmly fixed on the Mayfield clamp. Preoperative CT scans of the craniovertebral junction were registered for navigation. Surface contour registration was then done by a cloud of paired points by using the navigation probe and the infrared optical tracking system. The navigation accuracy was confirmed to be within 1 mm by anatomical marks. After removing the reference frame, the face, nasal cavity, and oral cavity were prepped with 0.5 % iodine. The Davis mouth gag was placed, and bilateral catheters were used to retract uvula to expose the posterior oropharyngeal wall. Using frameless navigation, the midline of the atlas and the base of the odontoid process were identified. The mucosa of the oropharyngeal wall was opened in the midline from the anterior arch of the atlas down to the vertebral body of the axis. The longus capitis and the longus colli muscles were dissected subperiosteally from the midline on both sides to the medial edges of atlantoaxial joints. Under the guidance of navigation, important landmarks were identified, such as the tip and the lateral edge of the odontoid process, and this helped to determine the extent of bony resection (Fig. 2). All patients underwent microscopic transoral odontoidectomy assisted by navigation. The odontoid process and part of C2 vertebral bodies were drilled away in order to completely remove all bony compression of the spinal cord (Fig. 3). The tip of the odontoid process was often scarred to the dura, and the ligaments and scar tissue were dissected off with blunt dissection and with Kerrison rongeurs. An intraoperative CT scan was performed to confirm the extent of bony resection. After confirmation of adequate decompression, the surgical wound was closed in layers. A nasogastric tube was kept in place for 1 week after the operation. Tracheal intubation was removed on postoperative day 1. The patient was begun on a diet on postoperative day 5. Postoperative CT and MRI scans were obtained to assess the adequacy of CVJ decompression and to assess the fixation of the occipitocervical junction 1 week later.

## Results

Eleven patients had undergone the aforementioned procedure from December 2011 to March 2018. All patients were diagnosed with BI and AAD, six patients were associated with syringomyelia, and 4 had Chiari malformation. All patients had undergone a primary posterior distraction and fixation to decompress the spinal cord by distracting the odontoid process from the cranium. One patient had already undergone a prior anterior transoral decompression and posterior instrumentation at another hospital. Due to incomplete decompression or progressive neurological deterioration, all patients underwent a microscopic transoral odontoidectomy.

The mean time from the primary posterior surgery to transoral odontoidectomy was 3.8 years, ranging from 3 months to 8 years. The most common complaint of these patients was a progressive weakness of the upper and lower extremities (10 patients, 90.9%). Paresthesia occurred in 9 patients (81.8%), and neck pain occurred in 8 patients (72.7%).

The mean follow-up time was 47 months, ranging from 4 to 81 months. Nine patients had significant symptom relief, and 1 patient remained stable. No neurological deterioration after surgery was observed. The mean operative time was 174.1 min, and the mean blood loss was 85.5 ml. The mean JOA score improved from 7.0 ± 0.9 to 11.4 ± 1.4 postoperatively (*p* < 0.01). The mean CMA improved from 119.3° ± 14.2°to 129.1° ± 11.1° (*p* < 0.01) (Table 1). One patient had pharyngeal mucosa dehiscence which required repair. No wound infection or cerebral spinal fluid leakage occurred.

**Fig. 4:**
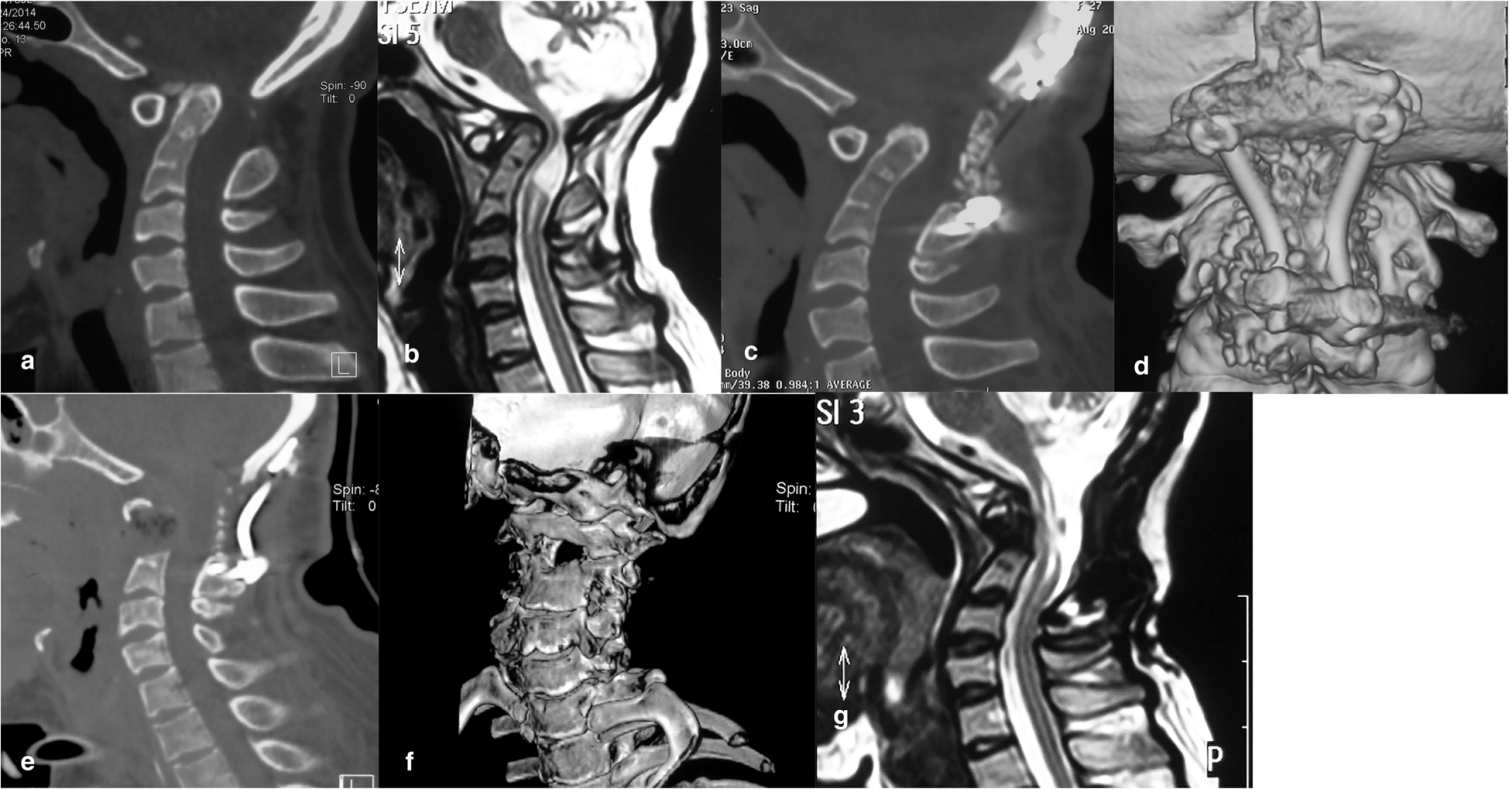
**a** Sagittal CT image and **b** sagittal MRI before the first posterior operation, suggesting that the patient had BI and AAD. The spinal cord was compressed from the ventral aspect. **c**, **d** Indicating that after posterior distraction and fixation, AAD reduction was not satisfactory, the occipital foramen is enlarged and bone graft fusion is solid. **e**, **f** Three months after the operation, CT images suggesting that the odontoid process was fully resected, part of C2 vertebral body was simultaneously resected, and the foramen magnum was further enlarged. **g** Sagittal MRI revealing decompression of the spinal cord

After the transoral odontoidectomy, CT and MRI images taken 1 week postoperatively confirmed complete odontoidectomy in 8 patients (Fig. 4), but incomplete odontoidectomy in 3 cases. A second revisional transoral odontoidectomy was performed in 2 cases, and one patient refused a second transoral odontoidectomy. One patient died of unknown causes 7 days after the surgery while asleep. The patient who died was a 31-year-old male, who underwent posterior AAD reduction and fixation 1 year prior. The symptoms did not abate after the posterior operation. The patient then underwent transoral odontoidectomy, and he recovered without incident after the surgery. His neurological symptoms improved and his vital signs were normal. The patient died of sudden death during sleep 1 week after the surgery.

## Discussion

The congenital or developmental bony anomalies at the CVJ are complex and primarily consist of AAD and BI [1, 18, 19]. These abnormalities may cause compression of the spinal cord or medulla, leading to myelopathy, intractable neck pain, and increased risk of worsening neurologic function due to trauma [18, 20]. The main aims of surgical treatments are relieving the cervicomedullary compression, restoring the stability of the CVJ, and restoring normal cerebrospinal fluid flow [18, 21].

Treatment of AAD with BI is still challenging because of abnormal osseous anatomy, and deformities of the facet joint make the reduction of AAD difficult with cervical traction, even under general anesthesia [20, 21]. After the introduction of the posterior distraction and fixation technique, the surgical treatment algorithm had changed from anterior transoral odontoidectomy followed by posterior instrumentation to posterior surgery only [1, 4, 13, 20, 22]. With intraoperative manipulation, spinal cord compression from the odontoid process could be alleviated by the reduction and distraction of the odontoid away from the brainstem and spinal cord. However, there were still cases with persistent neurological symptoms even after posterior reduction and indirect decompression. Possible reasons for inadequate decompression are as follows: (1) the complex bony abnormality hinders reduction of AAD or/ and BI, (2) severe obliquity of facet joint makes the placement of intrafacet spacer difficult or impossible, or (3) abnormal bony auto-fusion secondary to arthritis makes reduction impossible [6, 9, 20, 22]. Under such circumstances, a second stage transoral odontoidectomy could be considered for patients with residual medulla and cervical spinal cord compression.

The anterior approach through the posterior pharyngeal wall is the anatomically shortest path to the ventral aspect of the craniocervical junction [8, 10, 21]. The transoral odontoidectomy was once considered to be the gold standard for decompression at the craniovertebral junction, and this approach has its obvious advantages, such as providing direct access to the craniovertebral junction and avoiding critical anatomic structures ( e.g., Eustachian tube, internal carotid artery, and pterygoid nerve). In the past few decades, transoral odontoidectomy has proven to be a safe and effective surgical technique with low mortality in patients with irreducible AAD associated with Chiari malformation, displaced odontoid fractures, or BI [21]. In congenital anomalies, extremely abnormal osseous anatomy can make it difficult to confirm the midline of the odontoid process which could lead to incomplete odontoidectomy [22]. In BI/AAD patients, local distorted osseous anatomy could also lead to disorientation and asymmetrical odontoid drilling during the transoral procedure. Several studies have reported the use of navigation in the upper cervical spine to overcome this situation, showing good results and safety [23–25]. However, in our series, 3 patients (3/11) had incomplete odontoidectomy even with the help of navigation. Asymmetrical bony drilling toward the dural sac or oblique drilling of the odontoid process may lead to an erroneous estimation of the completeness of the odontoidectomy. In order to address this problem, the intraoperative CT can be a very useful tool to precisely identify the position of the odontoid.

**Fig. 5:**
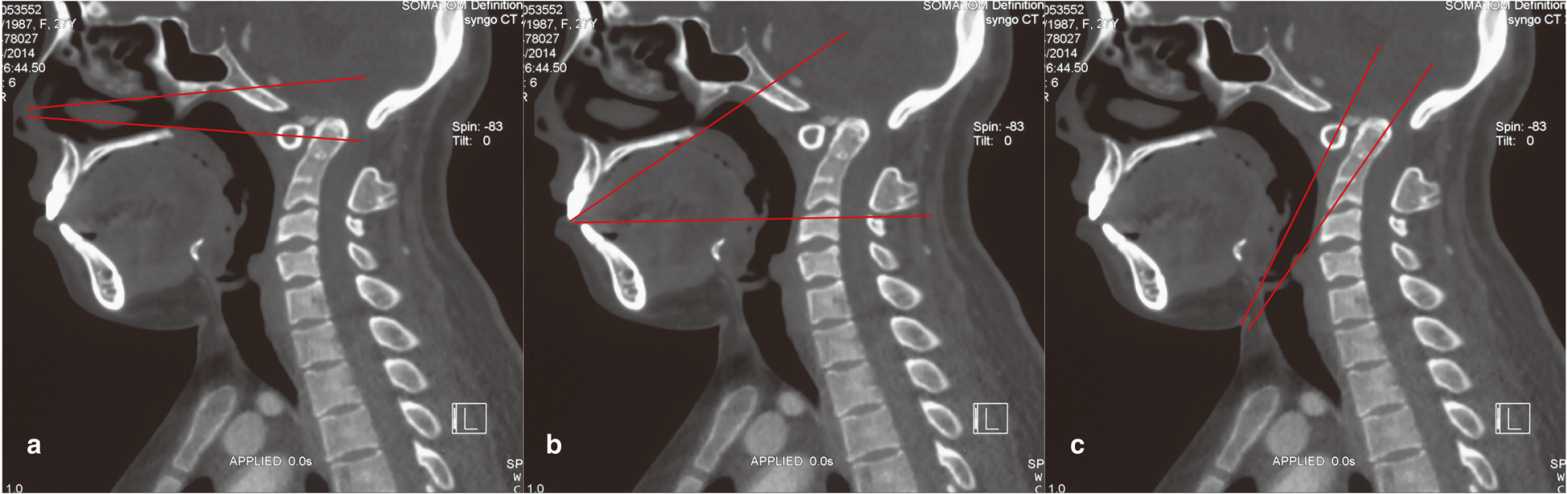
Showing the surgical corridor and the extent of exposure of three classic odontoid resection approaches in a patient with BI and AAD. **a** Transnasal, **b** transoral, and **c** transcervical

The endoscopic transnasal odontoidectomy, however, only requires an incision in the upper part of the pharynx, and the risk of infection may be less compared with the transoral approach. However, the endoscopic transnasal approach has obvious limitations. The range of exposure to the surgical target area is less, and the surgical instruments can only reach the upper edge of the axis, whereas C3 can be reached through the transoral approach (Fig. 5) [23–25]. Moreover, the surgical window of the transnasal approach is longer and narrower, thus making it more difficult in the manipulation of surgical instruments and demanding more skills from the surgeon [26]. Also, suturing the nasopharynx incision through nasal approach can be more difficult [27, 28]. In addition, if a cerebrospinal fluid leak occurs during surgery, the primary closure is very challenging or even impossible.

With regard to the CMA, Wang et al. reported the range of CMA in normal patients ranging from 139.0° to 175.5°, with an average of 158.5° [17]. The CMA is considered to be an effective index to assess the grade of anterior spinal cord compression. In CVJ malformation, especially BI/AAD, the CMA is usually lower than normal [5, 14]. In our series, the mean pre-operative CMA was 119.3° ± 14.2°, the medulla and the spinal cord were severely compressed. After odontoidectomy, the CMA improved to 129.1° ± 11.1° (*p* < 0.01). Because of the alleviation of ventral bony compression and the reduction of medullary-spinal cord kinking, the patient’s neurological symptoms also improved after surgery.

We previously reported a series of 135 AAD patients with a 3-year follow-up, and we proposed a treatment algorithm [13]. For both reducible and irreducible dislocation patients, direct posterior reduction and fixation procedure would first be attempted. For patients who had no effective decompression after attempted posterior reduction because of anatomic constraints, a postoperative MRI showed that there was still anterior spinal cord compression, and the patient’s clinical symptoms still persisted, a transoral odontoidectomy should be performed.

The application of navigation has been useful in both cranial and spinal surgeries because it provides three-dimensional spatial orientation during surgery [28–30]. When dealing with BI and AAD with abnormal anatomy, navigation affords higher precision and accuracy to help with the odontoidectomy. Navigation also decreases the need for intraoperative fluoroscopy, hence decreases the amount of radiation exposure both to the patient and to the operating room staff.

This study had several shortcomings and limitations such as lacking control group, having a relatively small sample size, and single-institution data. Due to the clinical complexity of this pathology, the heterogeneity of this patients population is relatively large, and the treatment options are also relatively complex. In many cases, the initial posterior surgery was performed at different hospitals, and some patients even have undergone multiple surgeries. Although these are limiting factors, this disease entity tends to be rare, especially in patients who have failed posterior distraction and stabilization.

One patient died suddenly of unknown causes 7 days after the surgery while asleep. We speculated that the patient may die of pulmonary embolism or respiratory failure, which could be caused by long-term compression of the medulla oblongata.

## Conclusion

Transoral odontoidectomy as salvage surgery is indicated in some selected patients with BI and AAD after posterior reduction and fixation failure. Transoral odontoidectomy is a relatively safe, technique-demanding, and effective salvage surgery in patients with inadequate posterior reduction and lack of improvement in clinical symptoms, which has a potential risk to damage the spinal cord and respiration center. Navigation assisted by intraoperative CT scan can safely guide the surgical procedure in the modern era, especially for irregular bony anatomy which is common in complex congenital CVJ malformation.

## Data Availability

All the data and materials of this series are available in the Neurosurgical Datasets of Xuanwu Hospital.

## Abbreviations

AAD: Atlantoaxial dislocation
B: Basilar invagination
CMA: Cervicomedullary angle
CT: Computed tomography
CVJ: Craniovertebral junction
JOA: Japanese Orthopedic Association
MRI: Magnetic resonance imaging
